# Observations of the Determination of the Hearing Power

**Published:** 1885-05

**Authors:** W. Laidlaw Purves


OBSERVATIONS OF THE DETERMINATION OF THE HEARING POWER. *By* W. LAIDLAW PURVES, M. D.

Despite the numerous proposals and methods of testing the acuteness of hearing in affections of the ear, the instruments to which surgeons almost exclusively confine themselves for that purpose are the watch and the tuning-fork. That with such, a complete examination cannot be made will be admitted by all who have paid attention to the subject. The want of an instrument by which the intensity of speech could be regulated, and of a method of determining the accommodation of the ear is still felt. That something in this direction may be arrived at by a modification of the telephone we may reasonably hope. Meantime I wish to direct attention to one or two points regarding the determination with the present instruments at our command.

In testing with the watch or other acoumeter of known intensity, the acuteness of hearing is determined by measuring the greatest distance at which the vibrations emitted by the instrument are heard through the air. Should that correspond to the distance already determined to be that of normal hearing, the hearing through the usual sound-conducting apparatus is considered normal. Should it be greater or less it is + or − and the amount is noted. The watch may also be employed for roughly testing the power of audition through the cranial bones by mapping out the positions on the cranium at which a certain watch is heard, but the tuning-fork is a much more convenient instrument for this purpose, as by it the intensity of the sound may be increased at will, within limits which suffices for our purposes, and the instant at which the perception of vibrations ceases can be more exactly stated.

The acuteness is here determined by striking a prong of the tuning-fork against some hard substance, and placing the extremity of the handle on some part of the cranium, the teeth, vertex, or mastoid process, usually noting the weakest vibration perceived by the patient, and then measuring the time the note continues to be heard by a normal ear after the transference of the fork to a corresponding part of the observer's head. This does not give us an exact result, but for usual clinical purposes it is sufficiently so. If the fork be transferred on the cessation of the auditory perception from the bone, and placed closely to the meatal opening, the sound will continue to be heard for some time longer should the conducting organs be in a normal condition. It is to the length of time which a normal ear continues to hear these aerially transmitted vibrations that I wish to draw attention.

The duration of the meatal after the mastoideal hearing is influenced by several factors, viz., the position of the fork on the cranium, the note of the fork, the size of the fork, and probably by other agents to which we need not here refer.

From the teeth the waves are conveyed to the auditory nerve, probably better than from any other part, but as they are not always present nor in a condition to be placed at the surgeon's service, and as the end to be attained can be reached more conveniently from the mastoid, it has been chosen as the spot through which perosseal audition is conveyed.

Causing a C = 512, with prongs 4½ in. in length, to vibrate, and placing it on the portion of the mastoid at which it is best heard, and then on the cessation of the perception through the osseous structures transferring it to the meatal orifice, I find that it continues to be heard by me for thirty seconds. On determining this with what are considered normal ears, I find this ranges from twenty-five to thirty-five seconds, depending partly on the age of the patient. Can we use this ratio of the meatal mastoideal hearing in any way for the more exact localization of affections, and the apportioning of the amount of loss to its proper seat, where both the conducting apparatus and the nerve are implicated?

If the tuning-fork ceases to be heard by the patient and a healthy observer at the same moment, we conclude roughly that the acoustics of both are equally sensitive to that tone. Should, however, the patient hear vibrations to which the observer's acoustic does not respond, we conclude that either the acoustic of the patient is more sensitive or that there is some additional reflection by some hindrance to the dispersion of wave-sounds from the ear. The number of seconds during which the vibrations are heard after they are lost to a healthy ear is noted as + per mastoid. To determine to which locality we must assign this, the duration of the meatal hearing after the cessation of the mastoideal is determined. Should this also be above the normal standard a hypersensitiveness of the acoustic is determined. But should the meatal hearing be of less than the normal duration after the mastoideal has ceased, we conclude that vibrations to which the acoustics ought to respond through the meatus are not of sufficient strength to set in motion the conducting apparatus, and we have thereby a gauge as to the amount of obstruction to the passage of vibrations through the normal channels.

Should the acoustic be deficient but the meatal hearing be in the same proportion to the mastoideal, the conducting media may be taken as normal, and the deficiency of the acoustic taken by the number of seconds during which a healthy nerve detects the pulsations after the removal of the fork to the mastoid of the observer. Or, by closing the meatus of the patient, and so causing an increase to and perception of the vibrations by the deficient acoustic as long as they are detected by a healthy nerve through the mastoid, the difference of the meatal hearings thereafter will give the deficiency.

To make these and many such like determinations conveniently, a stop-watch, a large fork, and an apparatus formed to cause the fork to be heard by the patient and by the observer at the same time and with equal pressure, are required. By these the physician may determine the functional power of the acoustic, and without visually examining the meatus and cavity assign in a general way the loss, if any, to its proper position. The determination, as generally carried out, must lead to false conclusions if the difference of the meatal from the mastoideal hearing, and the ratio of the two are not kept constantly in view. We require numerous observations before we can fix a standard of the two hearing powers at different ages and of the proportion of one to the other, but having gained these, we shall have added another step to the exactitude of our diagnosis. That many acoustic changes which are not detected occur in early stages of different nervous diseases I feel certain, and I would assure neurologists that one hour's application would enable them to add a valuable indication to their means of detection of early abnormal deviations.—*Guy's Hospital Reports Volume 42.*

